# DRG2 Accelerates Senescence via Negative Regulation of SIRT1 in Human Diploid Fibroblasts

**DOI:** 10.1155/2021/7301373

**Published:** 2021-11-03

**Authors:** Bing Si Li, Ai Lin Jin, ZiQi Zhou, Jae Ho Seo, Byung-Min Choi

**Affiliations:** ^1^Department of Biochemistry, Wonkwang University School of Medicine, Iksan, Jeonbuk 54538, Republic of Korea; ^2^Department of Herbology, Wonkwang University School of Korean Medicine, Iksan, Jeonbuk 54538, Republic of Korea; ^3^Sarcopenia Total Solution Center, Wonkwang University School of Medicine, Iksan, Jeonbuk 54538, Republic of Korea

## Abstract

Accumulating evidence suggests that developmentally regulated GTP-binding protein 2 (DRG2), an evolutionarily conserved GTP-binding protein, plays an important role in regulating cell growth, inflammation, and mitochondria dynamics. However, the effect of DRG2 in aging remains unclear. In this study, we found that endogenous DRG2 protein expression is upregulated in oxidative stress-induced premature senescence models and tissues of aged mice. Ectopic expression of DRG2 significantly promoted senescence-associated *β*-galactosidase (SA-*β*-gal) activity and inhibited cell growth, concomitant with increase in levels of acetyl (ac)-p53 (Lys382), ac-nuclear factor-kB (NF-*κ*B) p65 (Lys310), p21*^Waf1/Cip1^*, and p16*^Ink4a^* and a decrease in cyclin D1. In this process, reactive oxygen species (ROS) and phosphorylation of H2A histone family member X (H2A.X), forming *γ*-H2A.X, were enhanced. Mechanistically, ectopic expression of DRG2 downregulated Sirtuin-1 (SIRT1), resulting in augmented acetylation of p53 and NF-*κ*B p65. Additionally, DRG2 knockdown significantly abolished oxidative stress-induced premature senescence. Our results provide a possible molecular mechanism for investigation of cellular senescence and aging regulated by DRG2.

## 1. Introduction

Aging is one of the biggest risk factors for the development of various diseases, including dementia, chronic respiratory diseases, angiocardiopathy, infection, and cancer [1]. At cellular and molecular levels, senescent cells play important roles in tissue deterioration and disorganization and in organ dysfunction. Reduction of senescent cell levels is related to a significant decrease in the incidence of aging-associated ailments, such as cardiovascular diseases [2, 3]. However, augmentation of senescent cells suppresses the development of cancer [4]. Cellular senescence was described first by Hayflick in the 1960s as an irreversible process of cell cycle arrest [5]. Cellular senescence exists spontaneously *in vivo* and *in vitro* and can be induced *in vitro* when cells are exposed to oxidative stress, such as hydrogen peroxide (H_2_O_2_) [5–7]. Senescent cells show several dramatic changes, including upregulation of structural proteins that allow enlarged and flattened cell morphology, increased senescence-associated *β*-galactosidase (SA-*β*-gal) activity, and secretion of proinflammatory cytokines [1]. The senescence process is accompanied by abundant accumulation of reactive oxygen species (ROS), which can result in severe damage to DNA, protein, and lipids [8].

Sirtuin-1 (SIRT1) is a nicotinamide adenine dinucleotide- (NAD+-) dependent class III histone deacetylase that takes part in numerous vital signaling pathways, such as those of DNA damage, apoptosis, mitochondrial biogenesis, cellular senescence, and inflammation [9]. Dysregulation of SIRT1 activity participates in aging-related diseases, including Alzheimer's disease, cardiovascular disease, neurodegeneration, obesity, and metabolic disorders [10]. SIRT1 expression is downregulated in chronic inflammatory conditions and the aging process, both of which involve oxidative stress [11]. SIRT1 localizes in the nucleus and deacetylate histones and nonhistone proteins, such as p53 and p65 (also known as RelA), a subunit of nuclear factor-kB (NF-*κ*B) [12–15]. SIRT1 deacetylates p53 and antagonizes p53-induced cellular senescence in response to DNA damage or oxidative stress, and SIRT1-deficient mouse embryonic fibroblasts (MEFs) exhibit hyperacetylation following DNA damage [13, 16]. SIRT1 limits inflammation by deacetylating p53 and p65, whereas inhibition of SIRT1 downregulates deacetylation and promotes activation of p53 and p65, leading to increased proinflammatory gene expression [14]. These observations suggest modulation of SIRT1 expression level as a potential treatment for aging and aging-associated diseases.

The small GTPase superfamily, one of the GTP-binding protein superfamilies, regulates many processes in eukaryotic cells such as signal transduction, cell proliferation, cytoskeletal organization, and intracellular membrane trafficking [17]. Recently, researchers found a novel subfamily of the GTPase superfamily, developmentally regulated GTP-binding proteins (DRGs), which has two closely related proteins, DRG1 and DRG2 [18–20]. DRG2 has been reported to control cell growth and differentiation, regulate mitochondrial morphology, and modulate inflammatory response [21–24]. Thus, we hypothesized that DRG2 affects the modulation of cellular senescence and aged tissue.

Here, we report the effect of DRG2 expression induced by H_2_O_2_ exposure or pcDNA-hDRG2 transfection in the WI-38 cell line. To elucidate the mechanism of DRG2 in cellular senescence, we explored the relationship between DRG2 and SIRT1. We further examined the effect of DRG2 knockdown on oxidative stress-induced premature senescence. Furthermore, we examined DRG2 expression in several organ tissues from C57BL/6 aged mice *in vivo*.

## 2. Materials and Methods

### 2.1. Reagents

Hydrogen peroxide (H_2_O_2_, #MKBK8393V) and paraformaldehyde solution (#SZBC2290V) were purchased from Sigma-Aldrich. 5-Bromo-4-chloro-3-indolyl-*β*-D-galactosidase (X-gal, 11680293001) was purchased from Roche. Antibodies against the following proteins were used: DRG2 (Proteintech group, 14743-1-AP), acetyl-p53 (ac-p53, Cell Signaling Technology (CST), #2525), p53 (CST, #2524), p21^Waf1/Cip1^ (Santa Cruz Biotechnology (SCBT), sc-397), cyclin D1 (SCBT, sc-717), p16^INK4*α*^ (BD Pharmingen, 551163), SIRT1 (Merck Millipore, 07-131), SIRT1 (Abcam, ab110304), acetyl-NF-*κ*B p65 (ac-NF-*κ*B p65, CST, #3045), NF-*κ*B p65 (SCBT, sc-8008), phosho-Histone H2A.X (p-H2A.X, CST, 9718 s), and *β*-actin (SCBT, sc-1616). Secondary antibodies for immunoblotting were from SCBT (goat anti-mouse IgG-HRP, sc-2005; mouse anti-rabbit IgG-HRP, sc-2357; donkey anti-goat IgG-HRP, sc-2020), and those for immunofluorescent staining were from Invitrogen (goat anti-rabbit Alexa Fluor 568, A-11011; goat anti-mouse Alexa Fluor 488, A-11001; goat anti-mouse Alexa Fluor 568, A-11004). DMF (6,4′-dihydroxy-7-methoxyflavanone) was obtained from the Standardized Material Bank for New Botanical Drugs (no. NNMBP012), Wonkwang University (Republic of Korea). DMF (>98%) was isolated from the heartwood of *Dalbergia odorifera* [25] and was dissolved in DMSO (0.05% in final culture concentration).

### 2.2. Cell Culture

Human embryonic lung fibroblasts (WI-38 cells, ATCC CCL-75) were bought from American Type Culture Collection (ATCC, Manassas, VA, USA). The cells were grown in Eagle's Minimum Essential Medium (EMEM; ATCC) containing 10% fetal bovine serum (FBS; Gibco BRL, Grand Island, NY, USA) and 1% penicillin streptomycin (10378016; Gibco) and stored in a humidified incubator (37°C, 5% CO2). WI-38 cells were seeded in a 60 mm dish or 6-well plate and cultured for 24 h before use in the following experiment.

### 2.3. Senescence-Associated *β*-Galactosidase (SA-*β*-Gal) Staining Assay

SA-*β*-gal staining was determined as described previously [26]. Aged cells (blue) and total cells were counted under fluorescence microscopy using a light microscope (Olympus, Tokyo, Japan).

### 2.4. Western Blot Analyses and Immunoprecipitation (IP)

The cells were harvested and resuspended in cold RIPA buffer (50 mM Tris-HCl buffer, pH 7.5, 150 mM NaCl, 1 mM EDTA, 1% NP-40, 2 mM phenylmethanesulfonyl fluoride, 5 mM protease inhibitor cocktail, and 1 mM Na orthovanadate). Protein concentration was quantified using a Bio-Rad proteins assay kit. Western blot analysis was performed as previously described [26]. For IP analyses, after sonication, whole cell lysates were precleared by preclearing with recombinant protein G agarose (15920-010; Invitrogen) slurry. The protein samples were added to 5 *μ*l SIRT1 antibody, 5 *μ*l DRG2 antibody, and 5 *μ*l normal IgG, respectively, and incubated overnight at 4°C using a rotator. Recombinant protein G (rProtein G) agarose (15920010; Invitrogen) was added to capture the immunocomplex for 4 h at 4°C with mixing. The immunocomplex was subjected to western blot analyses.

### 2.5. MTS Assay for Cell Proliferation

Cell viability was determined using a commercially available kit named CellTiter 96 ® AQ_ueous_ One Solution Cell Proliferation Assay kit (G3580; Promega). The assay was performed according to the manufacturer's instructions.

### 2.6. Plasmids, shRNA, and Transfections

The pcDNA6-V5/hDRG2 (human DRG2), pEGFP-N1/DRG2, and PLKO/ShDRG2 plasmids were obtained from the Department of Biological Sciences, Ulsan University (Republic of Korea). The pcDNATM4/his-Max A/hSIRT1 (human SIRT1) was obtained from the Department of Biological Sciences, Wonkwang University (Republic of Korea). These plasmid constructs have been described previously [24, 27]. Cells were transfected using Lipofectamine 2000 (11668-019; Invitrogen) according to the manufacturer's protocol (Invitrogen, Carlsbad, CA, USA). After transfection, the medium was changed, and the cells were used in other experiments.

### 2.7. Measurement of Reactive Oxygen Species (ROS) Level

Intracellular ROS staining was performed with chloromethyl 2′,7′-dichlorodihydrofluorescein diacetate (CM-H_2_DCFDA) according to the manufacturer's protocol (C6827; Invitrogen, Eugene, OR, USA). Briefly, cells were harvested and incubated in 10 *μ*M CM-H2DCFDA for 30 min at 37°C. After washing twice with cold PBS, cells were resuspended in FACS solution and then analyzed for fluorescence intensity using a FACS flow cytometer (FACSCalibur; BD Biosciences, San Jose, CA, USA).

### 2.8. SIRT1 Deacetylase Activity Assay

SIRT1 deacetylase activity was measured using a commercial kit (CS1040; Sigma-Aldrich) following the manufacturer's protocol [28]. The fluorescence signal was measured at excitation/emission wavelengths of 355/460 nm using a SpectraMax M3 instrument. Finally, the SIRT1 deacetylase activity was calculated using a standard curve.

### 2.9. Immunofluorescent Staining In Vitro

Cells were grown on glass coverslips and treated with H_2_O_2_ or transfected with plasmid for 72 h. Cells were washed with PBS and fixed with 4% paraformaldehyde for 20 min at room temperature (RT). After washing with PBS twice, the cells were permeabilized with ice cold 0.2% Triton X-100 (T8787; Sigma-Aldrich) for 5 min and then blocked with 3% Normal Goat Serum (#31872; Thermo Fisher) for 30 min at RT. Then, the cells were incubated with anti-DRG2 (1 : 250, rabbit; Proteintech), anti-SIRT1 (1 : 250, mouse; Abcam), and p-H2A.X (1 : 250, rabbit; CST) overnight at 4°C, washed twice with PBS, and incubated with Alexa Fluor 568 goat anti-rabbit antibody (1 : 1,000), Alexa Fluor 488 goat anti-mouse antibody (1 : 1,000), and Alexa Fluor 568 goat anti-mouse antibody (1 : 1,000) for 30 min at RT, respectively. The cells were stained with 4′6-diamidino-2-phenylindole (DAPI) (P36931; Sigma-Aldrich), washed with PBS twice, mounted on glass slides, and viewed on an Olympus FluoView 1000 confocal laser scanning system (Olympus, Tokyo, Japan).

### 2.10. Reverse Transcription-Quantitative Polymerase Chain Reaction (RT-qPCR)

Total RNA was extracted by easy-BLUE™ kit, according to the manufacture's protocol. RNA concentration was read using a SpectraMax® ABS Microplate Reader (Molecular Devices, San Jose, CA, USA). According to the manufacture's protocol, cDNA was synthesized with total RNA using ReverTra Ace® qPCR RT kit (TOYOBO, FSQ-101). The cDNA was mixed with IL-6 primer (Hs00174131_m1; Applied Biosystems; Thermo Fisher Scientific, Inc.) and then performed using Applied Biosystems™ StepOne™ Real-Time PCR System (LS4376357, Thermo Fisher Scientific, Inc.). Cycling conditions were performed as follows: preparation at 50°C for 2 min, denaturation at 95°C for 10 min, followed by 40 cycles at 95°C for 10 sec, and at 60°C for 30 sec. The data were analyzed using StepOne™ software (version 2.3; Applied Biosystems; Thermo Fisher Scientific, Inc.).

### 2.11. Animals

C57BL/6 male mice were obtained from the Central Laboratory Animal Inc. (Seoul, Korea). The animals were kept in a 12 h light/12 h dark cycle at 23 ± 1°C for 8 weeks or 24 months with free access to food and water. All animal studies were performed according to protocols approved by the Animal Care Committee of Wonkwang University (WKU15-18).

### 2.12. Immunofluorescent Staining In Vivo

Immunofluorescence assays for DRG2 were performed on paraffin-embedded muscle, heart, and liver tissue sections. The sections were incubated in different concentrations of ethanol and washed in PBS. The sections were boiled in an antigen retrieval buffer (10 mM sodium citrate buffer, pH 6.0, 0.5 ml Tween 20) for 10 min and washed in PBS. The sections were incubated in 0.3% H_2_O_2_ for 10 min at RT and blocked with 5% Normal Goat Serum for 1.5 h at RT. The sections then were incubated with primary antibodies against DRG2 (1 : 250 dilution in 5% Normal Goat Serum) overnight at 4°C, followed by the fluorescence-labeled secondary antibody Alexa Fluor 568 goat anti-rabbit (1 : 1,000). Nuclei were stained with DAPI (1 : 2,000) for 5 min at RT. The sections were mounted on glass slides and viewed on an Olympus FluoView 1000 confocal laser scanning system.

### 2.13. Statistical Analysis

All results were expressed as the mean ± standard error of the mean (SEM). One-way analysis of variance (ANOVA) analysis (R software) was used for data comparisons among groups. Values of *P* < 0.05 were considered statistically significant. The experiments were repeated three times under the same conditions.

## 3. Results

### 3.1. DRG2 Expression Is Upregulated in Oxidative Stress-Induced Premature Senescence in WI-38 Cells

**Figure 1:**
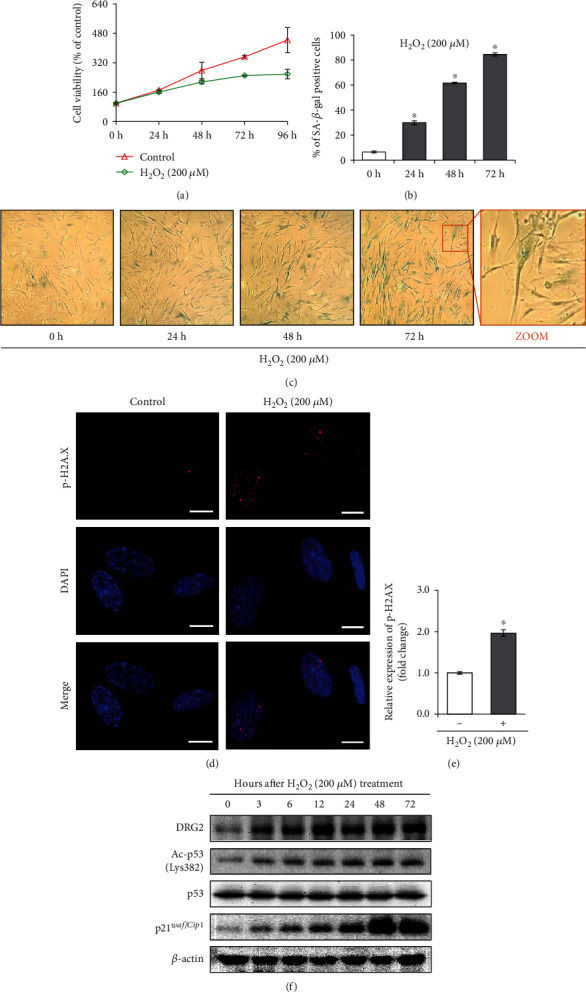
DRG2 is upregulated during H_2_O_2_-induced premature senescence in WI-38 cells. The cells were incubated in 200 *μ*M H_2_O_2_ and harvested at different time points. (a) Cell viability was determined by MTS assay. (b) and (c) The percentage of senescent cells was calculated from 3 random regions. Representative images of SA-*β*-gal staining of WI-38 cells (100× and 200× magnification). (d) and (e) Expression of p-H2A.X (red) was assessed by immunofluorescence staining, along with nuclear counterstaining using DAPI (blue) after H_2_O_2_ (200 *μ*M, 72 h) treatment. Scale bar, 10 *μ*m. Results shown were quantitated using Image J software. (f) Expression of DRG2, ac-p53 (Lys382), p53, p21*^WAF1/Cip1^*, and *β*-actin was analyzed by western blotting. Data are presented as the mean ± SEM value for each treatment. Similar results were obtained from three independent experiments. ^∗^*P* < 0.05 versus control.

Hydrogen peroxide (H_2_O_2_) is a well-known oxidative stress trigger for inducing cellular premature senescence [29, 30]. To build an oxidative stress-induced senescence model, cells were treated with 200 *μ*M H_2_O_2_ and were examined at various time points. We found that cell proliferation was significantly inhibited after 72 h and 96 h of H_2_O_2_ exposure (Figure 1(a)). The quantity of SA-*β*-gal stain-positive cells (blue) increased in a time-dependent manner with senescence-associated morphologic transformation to an enlarged and flattened shape (Figures 1(b) and 1(c)). The induction of SA-*β*-gal-positive cell staining reached a peak at 200 *μ*M H_2_O_2_ at 72 h. In addition, the phosphorylation level of H2A.X (*γ*-H2A.X), a DNA damage maker that usually accompanies cellular senescence [1], dramatically increased under fluorescence microscopy compared to that of the control group after H_2_O_2_ exposure (Figures 1(d) and 1(e)). Moreover, our further observations indicated that acetylation of p53 (Lys382) and p21*^Cip1/WAF1^*, two hallmarks of cellular senescence, augmented in a time-dependent manner after H_2_O_2_ exposure (Figure 1(f)). Parallelly, the protein level of DRG2 also increased gradually in WI-38 cells treated with 200 *μ*M H_2_O_2_ (Figure 1(f)). We speculated that DRG2 may correlate with H_2_O_2_-induced cellular premature senescence.

### 3.2. DRG2 Expression Accelerates Premature Senescence in WI-38 Cells

**Figure 2:**
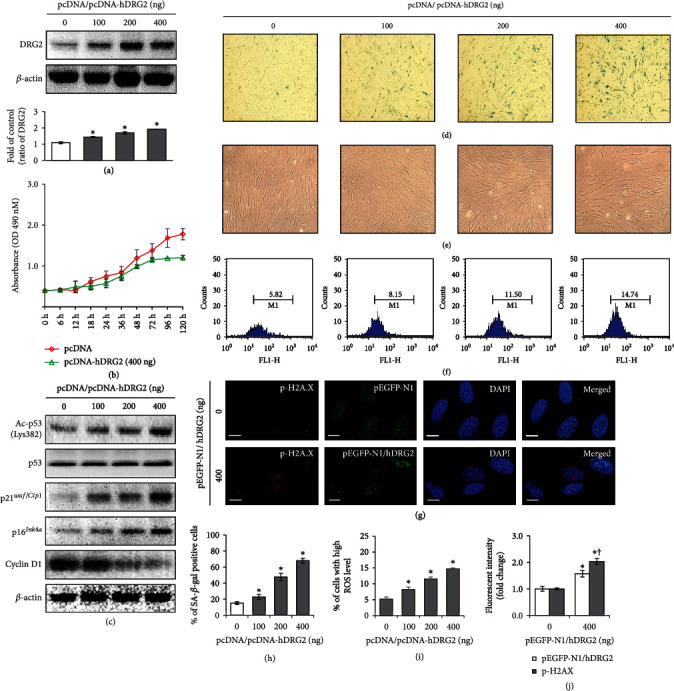
DRG2 is a positive regulator of cellular senescence. WI-38 cells were transfected with increasing doses (100, 200, and 400 ng) of pcDNA-hDRG2 plasmid or empty control for 18 h and grown in complete media for 72 h. (a) Expression of DRG2 and *β*-actin was analyzed by western blotting. DRG2 was quantified by densitometry based on immunoblot images. *β*-Actin was used as a loading control. (b) Cell proliferation was assayed by MTS assay. (c) Expression of ac-p53 (Lys382), p53, p21^WAF1/Cip1^, p16^Ink4*α*^, cyclin D1, and *β*-actin was analyzed by western blotting. (d) and (h) Representative images of SA-*β*-gal staining of WI-38 cells (40× magnification). The percentage of senescent cells was calculated from 3 random regions. (e) Representative images of cellular morphology. (f) and (i) ROS production was examined by CM-H_2_DCFDA fluorescent dye assay and analyzed by FACS. (g) and (j) WI-38 cells were transfected with pEGFP-DRG2 (400 ng) or empty control for 18 h and grown in complete media for 72 h. Expression of DRG2 (green) and p-H2A.X (red) was assessed by immunofluorescence staining, along with nuclear counterstaining using DAPI (blue). Scale bar, 10 *μ*m. Results shown were quantitated using Image J software. Data are presented as the mean ± SEM value for each treatment. Similar results were obtained from three independent experiments. ^∗^*P* < 0.05 versus control. ^†^*P* < 0.05 versus H_2_O_2_-treated cells.

To examine whether DRG2 overexpression promotes cellular premature senescence, different amounts of DRG2 expression plasmid (pcDNA/pcDNA-hDRG2) were transfected into WI-38 cells (Figure 2(a)). Ectopic expression of DRG2 decreased cell proliferation (Figure 2(b)). Next, we explored whether an increased level of DRG2 causes this alteration in cell proliferation by induction of senescence. DRG2 expression significantly increased senescent cell formation (blue) (Figure 2(d)) simultaneous with senescence-related morphological transformations (Figure 2(e)). The percentage of SA-*β*-gal activity increased in a dose-dependent manner (Figure 2(h)). Consistent with this result, increase in ac-p53 (Lys382), p21*^Cip1/WAF1^*, and p16*^Ink4a^* and decrease in cyclin D1 were observed (Figure 2(c)). Previous studies have reported that DNA damage and ROS accumulation are main effectors for acceleration of cellular premature senescence [31]. Therefore, we investigated whether DRG2 expression enhances alterations of DNA damage and ROS levels. As shown in Figures 2(f) and 2(i), DRG2 upregulation promoted ROS production maximally up to 14.74% at 400 ng of pcDNA-hDRG2 transfection. Also, we found upregulation of *γ*-H2A.X (red fluorescence) by overexpression of DRG2 (green fluorescence) (Figures 2(g) and 2(j)).

### 3.3. DRG2 Aggravates Oxidative Stress-Induced Premature Senescence by Suppressing SIRT1 Deacetylase Activity for p53

**Figure 3:**
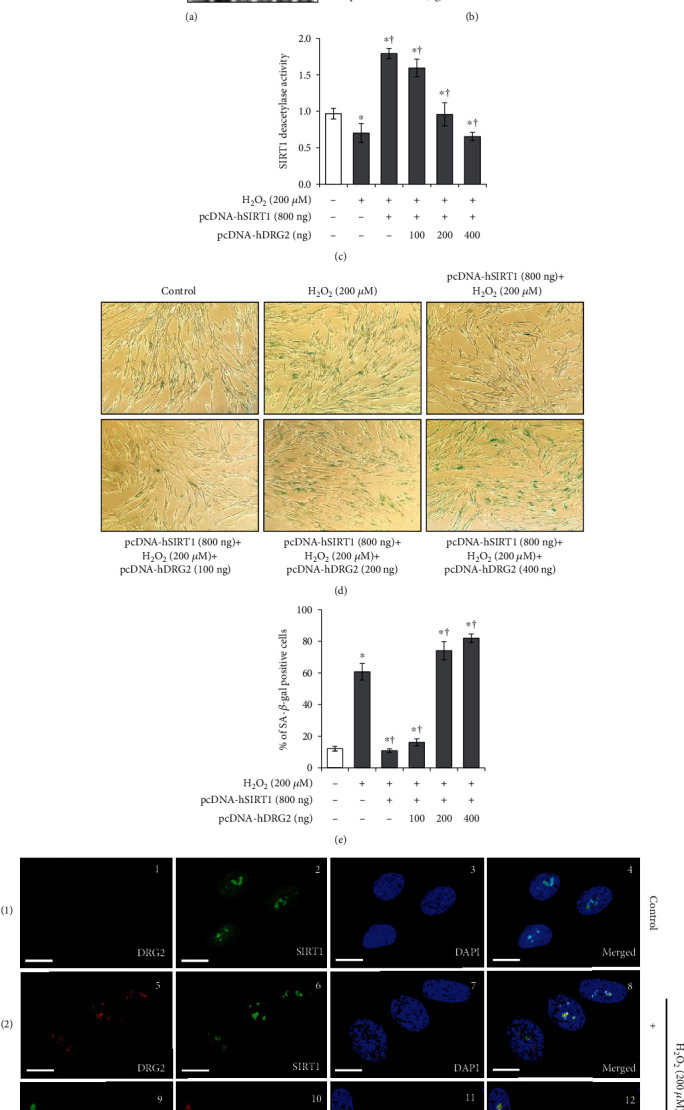
DRG2 inhibits SIRT1-mediated deacetylation of p53. (a)–(e) pcDNA-hSIRT1 (800 ng) was cotransfected into WI-38 cells with increasing doses (100, 200, and 400 ng) of DRG2-expressing plasmid or empty control for 18 h before exposure to H_2_O_2_ (200 *μ*M, 72 h). (a) Expression of SIRT1, ac-p53 (Ly382), p53, and *β*-actin was analyzed by western blotting. (b) Ac-p53 (Ly382) was quantified by densitometry based on immunoblot images. *β*-Actin was used as a loading control. (c) SIRT1 deacetylase activity was measured using a SIRT1 fluorescent activity assay. (d) and (e) Representative images of SA-*β*-gal staining of WI-38 cells (100× magnification). The percentage of senescent cells was calculated from 3 random regions. (f) (1-2) WI-38 cells were treated with or without H_2_O_2_ (200 *μ*M, 72 h). Expression of endogenous DRG2 (red) and SIRT1 (green) was assessed by immunofluorescence staining, along with nuclear counterstaining using DAPI (blue). (3) WI-38 cells were cotransfected to express both pEGFP-DRG2 (400 ng) and pcDNA-hSIRT1 (800 ng) plasmids for 18 h and grown in complete media for 72 h. Expression of DRG2 (green) and SIRT1 (red) was assessed by immunofluorescence staining, along with nuclear counterstaining using DAPI (blue). Scale bar, 10 *μ*m. Data are presented as the mean ± SEM value for each treatment. Similar results were obtained from three independent experiments. ^∗^*P* < 0.05 versus control. ^†^*P* < 0.05 versus H_2_O_2_-treated cells.

To clarify the molecular mechanism of how DRG2 drives cellular senescence, we focused on the dramatic increase in representative molecular markers (ac-p53) in cellular senescence. We considered whether DRG2 can control the function of SIRT1 because the SIRT1 plays a pivotal role in the regulation of cellular senescence by controlling p53 acetylation [15] and upregulation of SIRT1 or chemical activators for SIRT1 leads to the reduction of p53 acetylation [13, 28, 32]. To evaluate a role for DRG2-related signaling of SIRT1 in cellular senescence, we examined whether DRG2 can inhibit the deacetylase activity of SIRT1. Consistent with previous reports, augmentation of SIRT1 by pcDNA-hSIRT1 transfection abolished p53 acetylation mediated by H_2_O_2_ (Figure S1). Reversely, we transfected various amounts of the pcDNA-hDRG2 plasmid to examine the role of DRG2 on SIRT1 function. Our results showed that DRG2 overexpression reversed the effect of SIRT1 on p53 acetylation in a dose-dependent manner (Figures 3(a) and 3(b)). In our previous study, we found that 80 *μ*M of 6,4′-dihydroxy-7-methoxyflavanone (DMF) could effectively protect human diploid fibroblasts against H_2_O_2_-induced senescence by producing SIRT1 and inhibiting ac-p53 [32]. However, here, we found that DRG2 overexpression by pcDNA-hDRG2 plasmid reversed the effect of endogenous SIRT1 on p53 acetylation in a dose-dependent manner (Figure S2A and S2B). To confirm the effect of DRG2 on SIRT1 function, we measured SIRT1 deacetylase activity after overexpression of DRG2. Our data revealed that SIRT1 deacetylase activity was enhanced in SIRT1 overexpressed or DMF treated WI-38 cells, but DRG2 overexpression inhibited the suppressive effect of SIRT1 on p53 acetylation activity (Figure 3(c) and S2C). Moreover, the effect of cellular senescence, which was estimated by senescent-specific morphological changes and SA-*β*-gal activity in the same setting, indicated that DRG2 overexpression neutralizes the protective role of SIRT1 against H_2_O_2_-induced cellular senescence (Figures 3(d) and 3(e), S2D and S2E).

Next, we investigated the cellular localization of SIRT1 and DRG2 using confocal microscopy analysis. WI-38 cells were treated with or without H_2_O_2_ as a control. In the control group, SIRT1 was uniformly located in the nucleus (green fluorescent, Figure 3(f), panel 2). However, no visible colocalization was observed with DRG2 (Figure 3(f), panel 4). In the H_2_O_2_ treatment group, endogenous DRG2 (red fluorescent, Figure 3(f), panel 5) and SIRT1 (green fluorescent, Figure 3(f), panel 6) colocalized in the nucleus (Figure 3(f), panel 8). Next, WI-38 cells were cotransfected with pEGFP-DRG2 (green fluorescent) and pcDNA-hSIRT1 (red fluorescent). As expected, DRG2 and SIRT1 showed nuclear distribution (Figure 3(f), panel 9 and 10) and both proteins colocalized in the enlarged nucleus (Figure 3(f), panel 12), although we did not find any indication of direct interaction between DRG2 and SIRT1 via immunoprecipitation (Figure S3).

### 3.4. DRG2 Aggravates Oxidative Stress-Induced Premature Senescence by Suppressing SIRT1 Deacetylase Activity for NF-*κ*B p65

**Figure 4:**
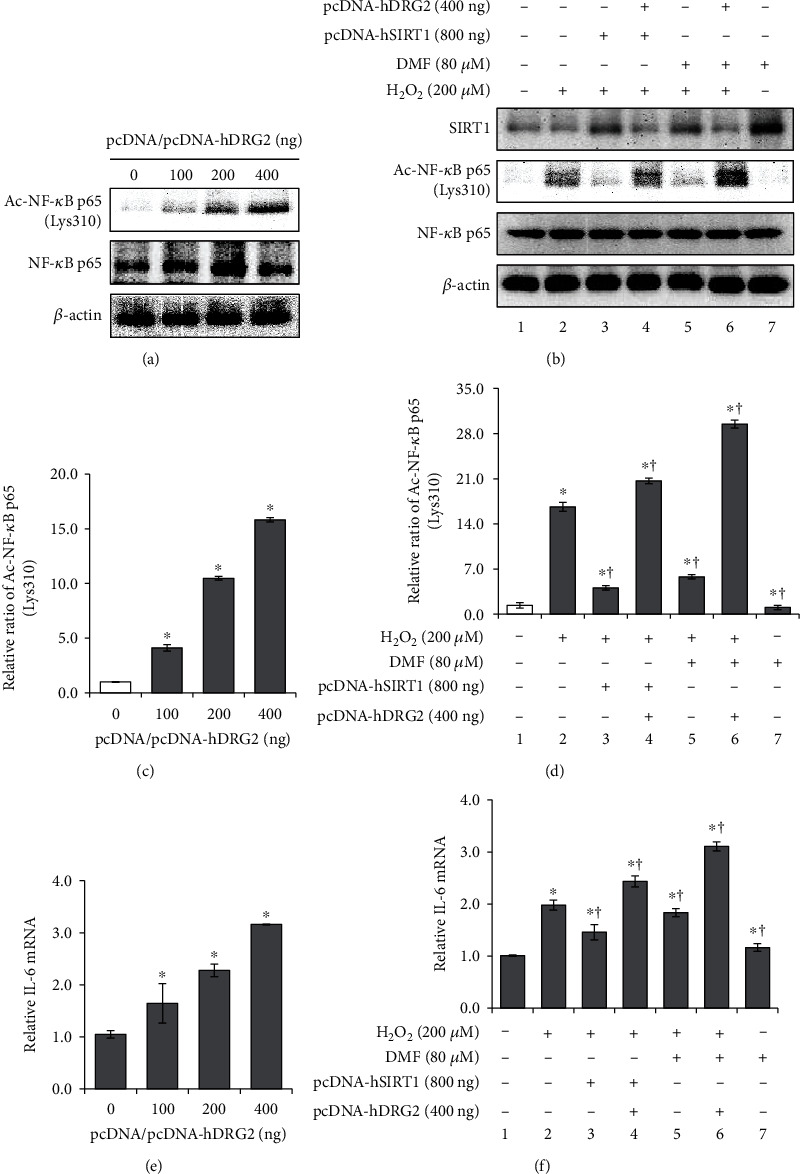
DRG2 inhibits SIRT1-mediated deacetylation of NF-*κ*B p65. (a) and (e) The cells were transfected with increasing doses (100, 200, and 400 ng) of DRG2-expressing plasmid or empty control for 18 h and grown in complete media for 72 h. (b) and (f) pcDNA-hSIRT1 (800 ng, lanes 3 and 4) was cotransfected into WI-38 cells together with empty control (400 ng, lane 3) or pcDNA-DRG2 (400 ng, lane 4) for 18 h. Empty control (400 ng, lanes 5 and 7) or pcDNA-DRG2 (400 ng, lane 6) was transfected into WI-38 cells for 18 h in the presence of DMF (80 *μ*M, 24 h, lanes 5-7). Then, the cells were incubated in H_2_O_2_ (200 *μ*M, 72 h, lanes 2-6). (a) and (b) Expression of SIRT1, ac-NF-*κ*B p65 (Lys310), NF-*κ*B p65, and *β*-actin was analyzed by western blotting. (c) and (d) Ac-NF-*κ*B p65 (Lys310) was quantified by densitometry based on immunoblot images. *β*-Actin was used as a loading control. (e) and (f) IL-6 mRNA was determined using RT-qPCR. Data are presented as the mean ± SEM value for each treatment. Similar results were obtained from three independent experiments. ^∗^*P* < 0.05 versus control. ^†^*P* < 0.05 versus H_2_O_2_-treated cells.

NF-*κ*B plays an important role in the inflammatory response, including enhancing the transcription of proinflammatory cytokines, which are closely associated with age-related diseases [33]. As DRG2 accelerated cellular premature senescence, we examined the effect of DRG2 on NF-*κ*B activation. DRG2 overexpression induced the expression of ac-NF-*κ*B p65 (Lys310) in a dose-dependent manner (Figure 4(a)). The level of acetylated NF-*κ*B p65 (Lys310) peaked at 14-fold that of the control at 400 ng of pcDNA-hDRG2 transfection (Figure 4(c)). Previous studies have shown that SIRT1 overexpression decreases acetylation of the RelA/p65 subunit of NF-*κ*B, followed by suppression of inflammation [11, 14]. As expected, SIRT1 induction by pcDNA-hSIRT1 or DMF significantly inhibited H_2_O_2_-induced ac-NF-*κ*B p65 expression (Figures 4(b) and 4(d), lanes 3 and 5). Based on this, DRG2 overexpression eliminated the deacetylation ability of SIRT1 for ac-NF-*κ*B p65 (Figures 4(b) and 4(d), lanes 4 and 6). NF-*κ*B regulates proinflammatory cytokines, such as IL-6 mRNA, which plays an important role in the modulation of senescence-associated secretory phenotype (SASP) [34, 35]. Our observations show that DRG2 overexpression increased IL-6 mRNA expression (Figures 4(e) and 4(f)), consistently with previous studies.

### 3.5. Knockdown of DRG2 Protects Cells against Oxidative Stress-Induced Premature Senescence

**Figure 5:**
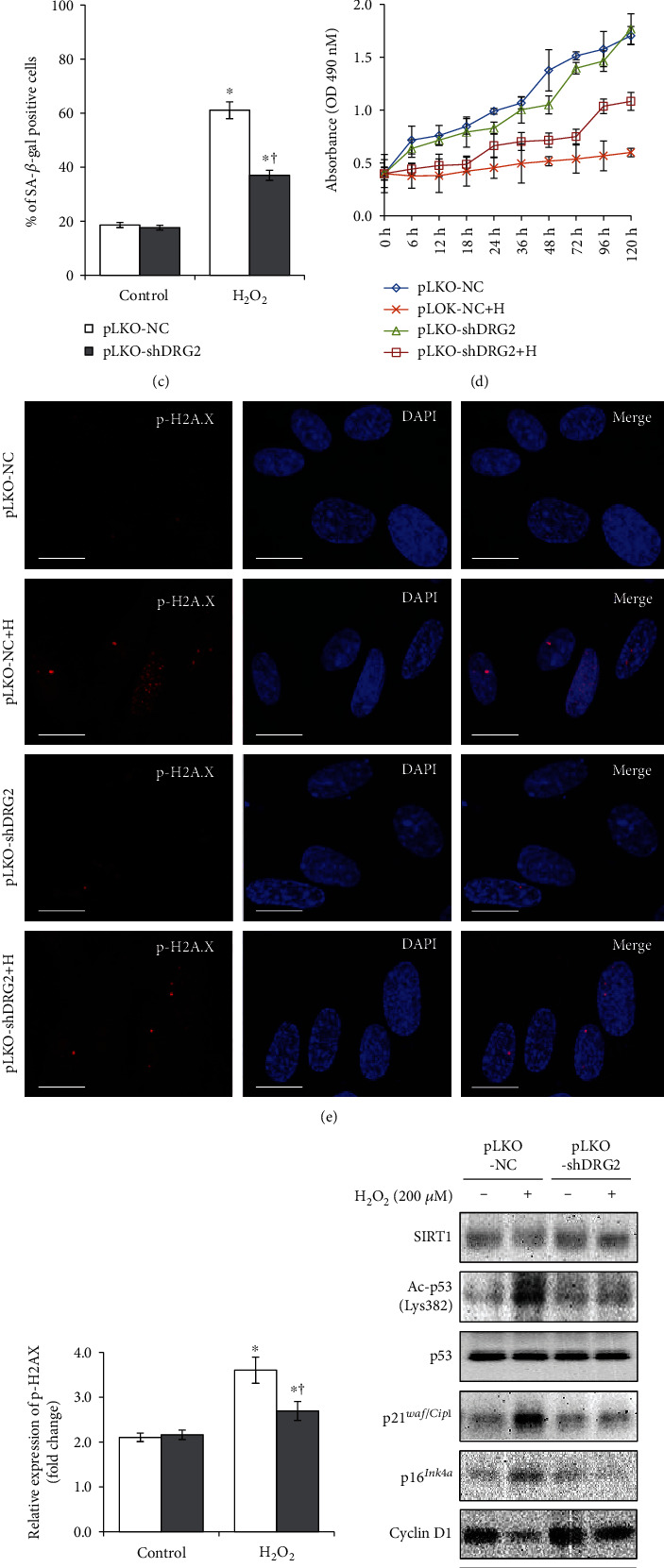
DRG2 knockdown protects cells from H_2_O_2_-induced premature senescence in WI-38 cells. The cells were pretransfected with pLKO-negative control (pLKO-NC) and pLKO-shDRG2 (400 ng, 18 h) in the presence or absence of H_2_O_2_ (200 *μ*M, 72 h). (a) Expression of DRG2 and *β*-actin was analyzed by western blotting. DRG2 was quantified by densitometry based on immunoblot images. *β*-Actin was used as a loading control. (b) and (c) Representative images of SA-*β*-gal staining of WI-38 cells (40× magnification). The percentage of senescent cells was calculated from 3 random regions. (d) Cell proliferation was assayed by MTS assay. (e) and (f) Expression of p-H2A.X (red) was assessed by immunofluorescence staining, along with nuclear counterstaining using DAPI (blue). Scale bar, 10 *μ*m. Results shown were quantitated using Image J software. (g) Expression of SIRT1, ac-p53 (Lys382), p53, p21^WAF1/Cip1^, p16^Ink4*α*^, cyclin D1, and *β*-actin was analyzed by western blotting. (h) SIRT1 was quantified by densitometry based on immunoblot images. *β*-Actin was used as a loading control. Data are presented as the mean ± SEM value for each treatment. Similar results were obtained from three independent experiments. ^∗^*P* < 0.05 versus control. ^†^*P* < 0.05 versus H_2_O_2_-treated cells.

To further verify whether DRG2 knockdown can block H_2_O_2_-induced senescence, WI-38 cells were transfected with pLKO-shDRG2 or pLKO-NC (negative control) in the presence or absence of H_2_O_2_. DRG2 knockdown was confirmed by western blotting (Figure 5(a)). DRG2 knockdown reduced SA-*β*-gal-positive cell staining (blue) (Figure 5(b)). SA-*β*-gal activity was reduced greatly in the DRG2 knockdown cells in the presence H_2_O_2_ (Figure 5(c)). Consistently, DNA damage signal (*γ*-H2A.X) by H_2_O_2_ was significantly reduced (Figures 5(e) and 5(f)), and cell proliferation was ameliorated in the DRG2 knockdown cells with H_2_O_2_-induced growth arrest (Figure 5(d)). Likewise, DRG2 knockdown abolished the suppressive of SIRT1 by H_2_O_2_ and inhibited the activation of its downstream molecules such as ac-p53 (Lys382), p21*^Waf1/Cip1^*, and p16*^Ink4a^* (Figures 5(g) and 5(h)).

### 3.6. DRG2 In Aged Muscle, Heart, and Liver

**Figure 6:**
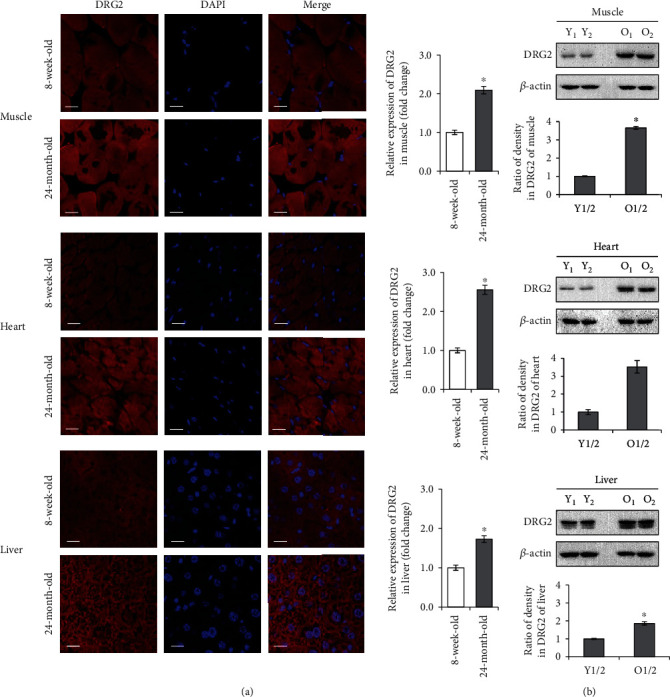
Immunofluorescence staining of DRG2 in aged mice. C57BL/6 male mice were sacrificed at 8 weeks old (Y_1/2_: young 1/2) or 24 months old (O_1/2_: old 1/2), and the muscle, heart, and liver were collected. (a) Expression of DRG2 (red) in sections was assessed by immunofluorescence staining, along with nuclear counterstaining using DAPI (blue). Results shown were quantitated using Image J software. (b) Expression of DRG2 and *β*-actin was analyzed by western blotting. The proteins were quantified by densitometry based on immunoblot images. *β*-Actin was used as a loading control. Data are presented as the mean ± SEM value for three mice in each group. Similar results were obtained from three independent experiments. ^∗^*P* < 0.05 versus control. Scale bar, 20 *μ*m.

To investigate the changes of DRG2 expression in young mice (8 weeks old) and aged mice (24 months old), we measured the expression level of DRG2 in the muscle, heart, and liver obtained from young and aged mice. Immunofluorescence (IF) staining for DRG2 was performed, and its relative expression was quantified (Figure 6(a)). DRG2 (red colour) was markedly increased in the aged mice. To confirm the above finding, the DRG2 protein expression was assessed by western blotting (Figure 6(b)). Consistently, DRG2 proteins increased in the aged mice tissues. These data indicate that the expression level of DRG2 in aged mice is much higher than that in young mice, and this *in vivo* observation strongly supports the proposed mechanism.

## 4. Discussion

**Figure 7:**
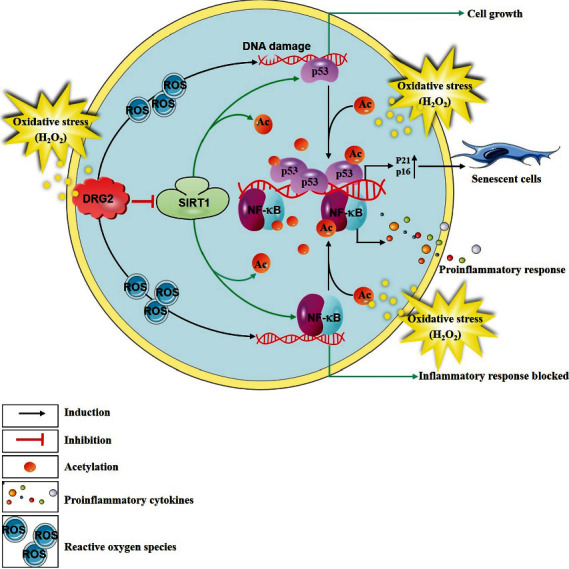
A schematic illustration summarizing the model for the role of DRG2 in cellular senescence. Upregulation of DRG2 promotes acetylation of p53 and NF-*κ*B p65 by inhibiting SIRT1 activity and protein level.

In this study, we showed that DRG2 is overexpressed in an H_2_O_2_-induced cellular senescence model, and that it regulates SIRT1 activity in an antiparallel manner in the cellular senescence process. This regulation plays a crucial role in balancing the acetyl modifications of p53 and NF-*κ*B p65 to switch on or off cellular senescence. Accordingly, the ectopic expression of DRG2 increases acetylation of p53 (Lys382) and NF-*κ*B p65 (Lys310), resulting in failure to upregulate SIRT1 expression and activity, and abrogate the protective effect of SIRT1 against H_2_O_2_-induced senescence (Figure 7). Moreover, DRG2 is upregulated in muscle, heart, and liver of aged mice *in vivo*.

Oxidative stress theory in aged was described first by Denhan Harman and is one of the most accepted hypotheses of molecular-level studies for aging [36–38]. Accumulation of chronic oxidative stress is produced by all cells of aerobic organisms owing to an imbalance between oxidant and antioxidant systems [39]. H_2_O_2_ has been used extensively as an inducer of oxidative stress in *in vitro* models [29, 30, 32]. We investigated DRG2 protein expression during H_2_O_2_-induced senescence in WI-38. Our results showed that DRG2 was upregulated after H_2_O_2_ exposure. Moreover, DRG2 was upregulated consistently in aged tissue from naturally aged mice.

Senescent cells exhibit an enlarged and flattened morphology, SA-*β*-gal activity, cell proliferation inhibition, ROS production, and alterations in expression of certain genes [1]. Therefore, we explored the role of DRG2 in the cellular senescence process. DRG2 overexpression by pcDNA-hDRG2 plasmid transfection strongly triggered inhibition of cell growth via upregulation of p53, p21^WAF1/Cip1^, and p16^Ink4*α*^ and downregulation of cyclin D1 with increasing SA-*β*-gal-positive signals, ROS, and *γ*-H2A.X in WI-38 cells. Although a previous study concluded that DRG2 knockdown substantially reduces growth speed but upregulates p21 protein level in HeLa cells [40], the role of DRG2 should be reconsidered because it can be induced in an H_2_O_2_-induced senescence model in WI-38 cells. Consistent with these reports, DRG2 overexpression suppresses cell growth in human T cells and reduces sensitivity to nocodazole-stimulated apoptosis [21, 22].

SIRT1 is a longevity-related gene that plays an important role in the regulation of inflammation and cellular senescence [11]. Previous studies have shown that SIRT-deficient cells exhibit hyperacetylation of p53 after DNA damage, but SIRT1-overexpressed cells sufficiently deacetylate p53 and block PML/p53-induced senescence [13, 16]. Han et al. reported that peroxisome proliferator-activated receptor-*γ* (PPAR*γ*), a ligand-regulated modular nuclear receptor, directly interacts with SIRT1 and inhibits SIRT1 activity in cellular senescence [41]. Ko et al. reported that DRG2 interacts with PPAR*γ* in antigen presenting cells, and this process enhances PPAR*γ* activity [24]. Thus, we chose to consider the role of DRG2 on SIRT1 in the senescence process. We found that DRG2 expression by pcDNA-hDRG2 decreases SIRT1 protein level and deacetylase activity and increases acetylation of p53. We also observed that DRG2 and SIRT1 colocalize in the nucleus, although we could not show a direct interaction between them by biochemical experiments.

SIRT1 reduction leads to acetylation of NF-*κ*B p65 and forked box O (FOXO3), as well as modification of histones H3 and H4, resulting in the expression of pro-inflammatory, antioxidant, prosenescent, and proapoptotic genes that are involved in inflammation, oxidative stress, and premature cellular senescence [11, 42]. Our data indicate that DRG2 expression induces NF-*κ*B p65 acetylation by suppression of SIRT1 expression and activity.

Interestingly, we also found that DRG2 knockdown strongly reduced senescence markers that respond to oxidative stress including p53, p21^WAF1/Cip1^, p16^Ink4*α*^, *γ*-H2A.X, and SA-*β*-gal activity under the condition induced cellular senescence by H_2_O_2_. Consistent with this, our observation shows that DRG2 level increased in naturally aged mouse tissues as well as the cells induced cellular senescence by H_2_O_2_. In this study, we propose that elevated level of DRG2 could induce premature aging and aging-related diseases.

## 5. Conclusion

In summary, this study demonstrated a previously unknown role for DRG2 in cellular senescence. DRG2 overexpression promoted premature senescence in normal cells and downregulated SIRT1 expression (Figure 7). In addition, we showed that downregulated DRG2 expression abolished oxidative stress-induced senescence. Consider that aging is a vital risk factor for aging-related diseases, and our study provides a possible new therapeutic strategy.

## Data Availability

The data used to support the finding of this study are available from the corresponding author upon request.

## Supplementary Materials

Supplementary Materials Figure S1: SIRT1 overexpression inhibited H_2_O_2_-induced p53 acetylase activity. Figure S2: DRG2 inhibits SIRT1-mediated deacetylation of p53. Figure S3: DRG2 does not interact with SIRT1.
