# Supplementary material for: DRG2 Accelerates Senescence via Negative Regulation of SIRT1 in Human Diploid Fibroblasts
Source: Oxid Med Cell Longev. 2021 Nov 3;2021:7301373. doi: 10.1155/2021/7301373 (PMC8580627; doi:10.1155/2021/7301373)
Supplement: Supplementary Materials — Figure S1: SIRT1 overexpression inhibited H2O2-induced p53 acetylase activity. Figure S2: DRG2 inhibits SIRT1-mediated deacetylation of p53. Figure S3: DRG2 does not interact with SIRT1. [file 7301373.f1.docx]

**Supplementary Figure**


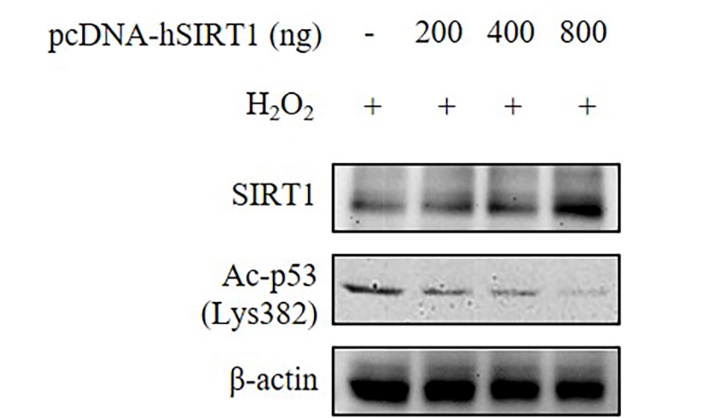


Supplementary Figure S1, Related to Figure 3: Performance of 800 ng pcDNA-hSIRT1 plasmid transfection significantly inhibited H_2_O_2_-induced p53 acetylase activity. WI-38 cells were transfected with increasing doses (200, 400, and 800 ng) of pcDNA-hSIRT1 plasmid or empty control for 18 h before exposure to H_2_O_2_ (200 μM, 72 h). Expression of SIRT1, ac-p53 (Lys382), and *β*-actin were analyzed by western blotting.


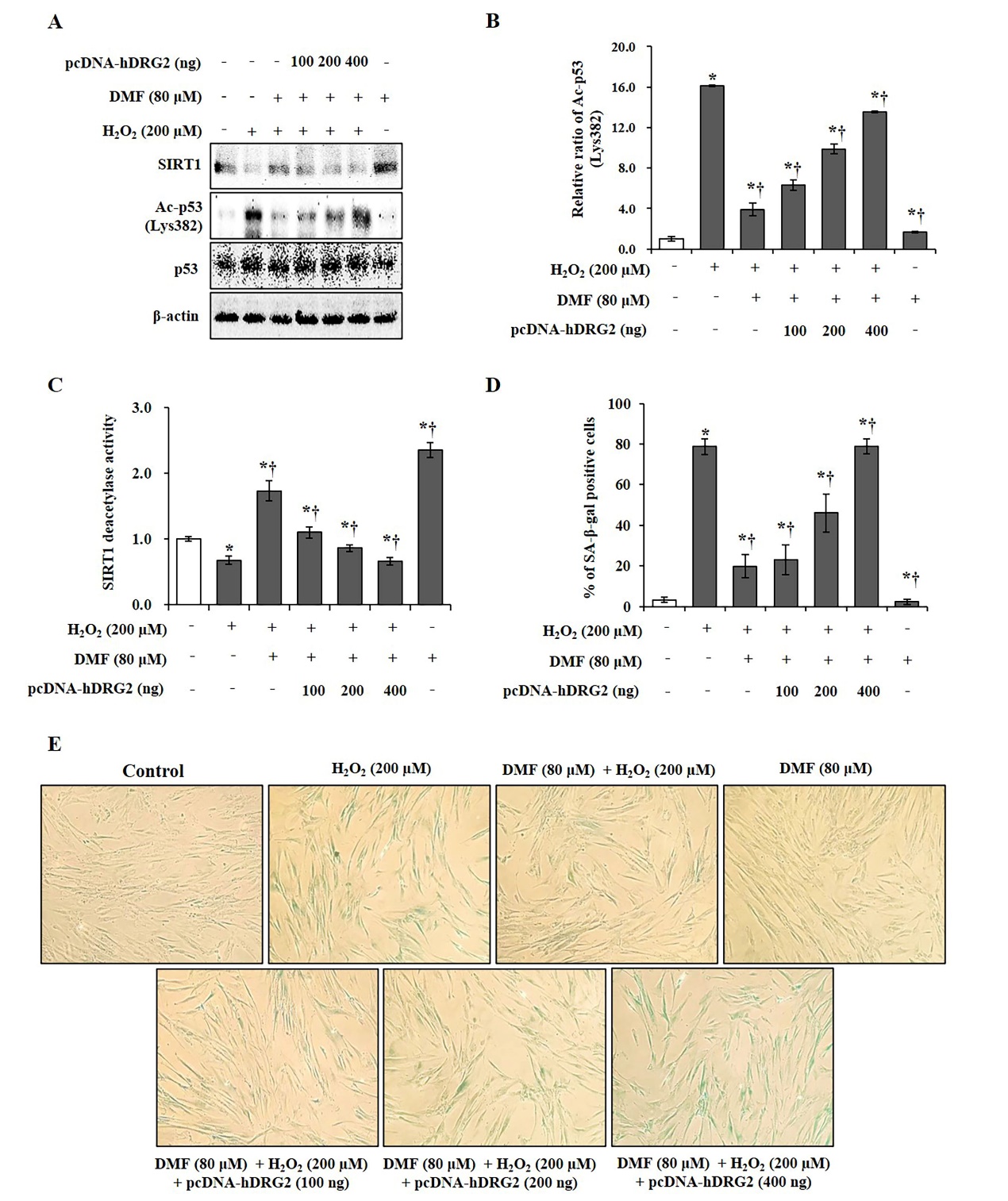
Supplementary Figure S2, Related to Figure 3: DRG2 inhibits SIRT1-mediated deacetylation of p53. WI-38 cells were transfected with increasing doses (100, 200, and 400 ng) of DRG2 expression or empty control for 18 h in the presence or absence of DMF (80 μM, 24 h) before H_2_O_2_ (200 μM, 72 h) treatment. (A) Expression levels of SIRT1, ac-p53 (Lys382), p53, and *β*-actin were analyzed by western blotting. (B) Ac-p53 (Lys382) was quantified by densitometry based on immunoblot images. *β*-actin was used as a loading control. (C) SIRT1 deacetylase activity was determined using a SIRT1 fluorescent activity assay. (D and E) The percentage of senescent cells was calculated from 3 random regions. Representative images of SA-*β*-gal staining of WI-38 cells (100× magnification). Data are presented as the mean ± SEM value for each treatment. Similar results were obtained from three independent experiments. * *P* <0.05 versus control. † *P* <0.05 versus H_2_O_2_-treated cells.


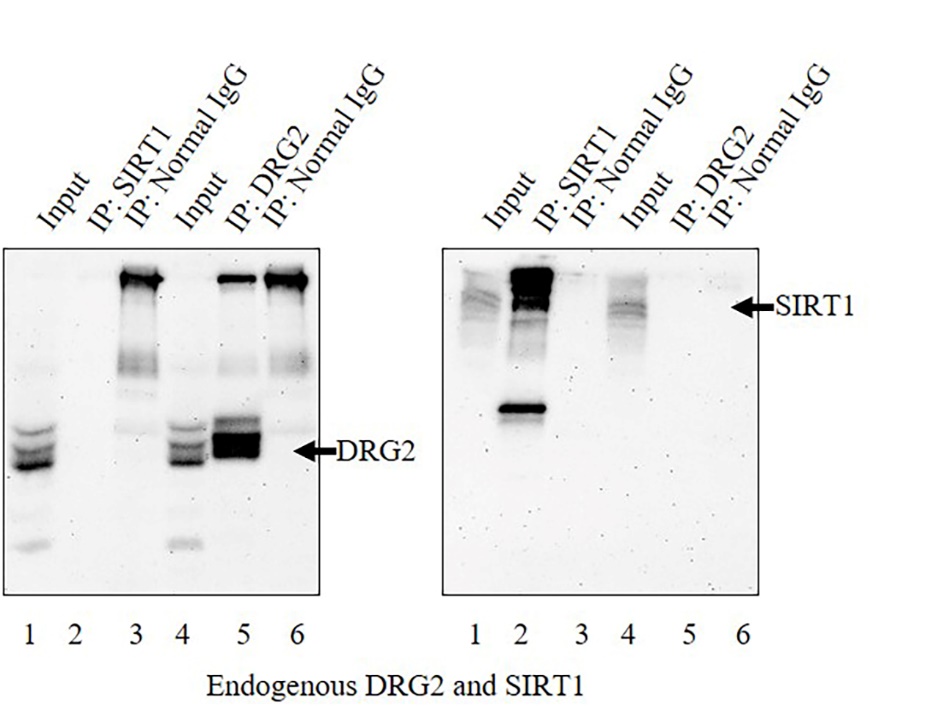
Supplementary Figure S3, Related to Figure 3: DRG2 does not interact with SIRT1. WI-38 cells were treated with H_2_O_2_ (200 μM, 72 h). Whole cell lysate was immunoprecipitated (IP) with anti-SIRT1 antibody (lane 2), anti-DRG2 antibody (lane 5), or control IgG (lanes 3 and 6). The immunocomplexes were analyzed by western blotting with anti-DRG2 (left) and -SIRT1 (right) antibody, respectively. DRG2 was not detected in the left lane (2). SIRT1 was not detected in the right lane (5). Normal Ig G as a negative control. Similar results were obtained from three independent experiments.
